# Inflammatory and Structural Endotypes of Human Atherosclerotic Plaque Revealed by Integrated Transcriptomic Analysis

**DOI:** 10.3390/genes17070779

**Published:** 2026-07-02

**Authors:** Eunseuk Lee, Anshu Sutihar, Meirajuddin Tousif, Song Peng Ang, Daniel Tran, Jose Iglesias

**Affiliations:** 1 Department of Internal Medicine, Community Medical Center, Toms River, NJ 08755, USA; anshu.sutihar@rwjbh.org (A.S.); meirajuddin.tousif@rwjbh.org (M.T.); 2 Division of Cardiology, Sarver Heart Center, University of Arizona, Tucson, AZ 85724, USA; songpengang@arizona.edu; 3 Department of Cardiology, Community Medical Center, Toms River, NJ 08755, USA; daniel.tran@rwjbh.org; 4 Department of Medicine, Hackensack Meridian School of Medicine, Nutley, NJ 07110, USA

**Keywords:** atherosclerosis, plaque, single-cell RNA sequencing, bulk RNA sequencing, transcriptomics, macrophages, smooth muscle cells, transcription factors, endotypes

## Abstract

Background/Objectives: Atherosclerotic plaque instability is driven by complex interactions among inflammatory, structural, and cellular remodeling programs. While bulk RNA sequencing provides insight into tissue-level transcriptional states and single-cell RNA sequencing (scRNA-seq) defines cellular heterogeneity, integration across these transcriptomic layers remains limited. We aimed to identify coordinated transcriptional programs associated with stable and unstable plaque phenotypes and map these programs to specific cellular compartments and regulatory networks. Methods: Paired bulk RNA-seq data from stable and unstable human carotid plaques (GSE120521) and scRNA-seq data from human coronary atherosclerotic lesions (GSE131778) were analyzed. Differential expression and Hallmark gene set enrichment analyses were performed using limma and clusterProfiler. Bulk-derived inflammatory and structural signatures were projected onto single-cell data using Seurat module scoring. Compartment-level transcriptional scores, an inflammatory–structural endotype index, and transcription factor activity inference using decoupleR and DoRothEA were used to characterize plaque-associated transcriptional states. Results: Unstable plaques demonstrated enrichment of inflammatory pathways, including interferon gamma response, inflammatory response, TNFα/NF-κB signaling, IL6/JAK/STAT3 signaling, complement activation, and reactive oxygen species pathways. In contrast, stable plaques demonstrated relative enrichment of myogenesis and structural remodeling programs. Projection of bulk-derived signatures onto single-cell data localized inflammatory programs predominantly to TREM2hi and inflammatory macrophage populations, whereas structural programs localized to smooth muscle cell and fibromyocyte-like compartments. Compartment-level analyses showed increased myeloid and adaptive immune signatures in unstable plaques and increased smooth muscle cell/fibro-remodeling signatures in stable plaques. Transcription factor activity analysis identified increased SPI1, NFKB1, RELA, and STAT1 activity in unstable plaques and higher SRF and TEAD1 activity in stable plaques. Conclusions: Integrative analysis of bulk and single-cell transcriptomic data identified distinct inflammatory and structural plaque transcriptional states associated with unstable and stable plaque phenotypes, respectively. These findings support a systems-level framework linking tissue-level plaque behavior to specific cellular and regulatory programs and provide evidence for inflammatory and structural plaque endotypes in human atherosclerosis.

## 1. Introduction

Atherosclerotic cardiovascular disease remains the leading cause of death worldwide and continues to impose a substantial global health burden despite major advances in preventive cardiology and lipid-lowering therapy [1,2]. Although age-adjusted cardiovascular mortality has declined in many high-income countries over recent decades, the absolute burden of ischemic heart disease continues to rise globally due to population aging and increasing prevalence of metabolic risk factors [3,4]. Acute coronary syndromes frequently arise not simply from progressive luminal stenosis, but from biologically vulnerable atherosclerotic plaques characterized by inflammatory activation and structural destabilization [5,6]. Histopathologic studies have demonstrated that rupture-prone plaques typically exhibit macrophage infiltration, necrotic core expansion, extracellular matrix degradation, and thinning of the fibrous cap, whereas more stable plaques are enriched in smooth muscle cells (SMCs), collagen deposition, and fibrotic remodeling [7].

The molecular pathways contributing to plaque instability have been increasingly characterized over the past two decades. Pro-inflammatory cytokine signaling through interferon-γ, TNF-α, IL-1β, and NF-κB pathways promotes macrophage activation, oxidative stress, and matrix metalloproteinase activity within unstable plaques, while the SRF–myocardin axis plays a central role in maintaining the contractile SMC phenotype associated with vascular stability [8,9,10,11]. However, despite growing knowledge of individual pathways and cellular populations, the systems-level organization of inflammatory and structural transcriptional programs across cellular compartments remains incompletely defined. In particular, it remains unclear whether plaque phenotype reflects the relative dominance of coordinated inflammatory versus structural transcriptional states operating across multiple interacting cell populations.

Recent advances in transcriptomic technologies have enabled increasingly detailed characterization of human atherosclerotic lesions. Bulk RNA sequencing studies have identified tissue-level inflammatory signatures associated with plaque progression and instability, while single-cell RNA sequencing (scRNA-seq) has revealed substantial cellular heterogeneity within plaque tissue [12,13,14,15,16,17]. Multiple independent scRNA-seq studies have identified TREM2hi macrophages, inflammatory monocyte-derived macrophages, modulated SMCs, fibromyocyte-like stromal cells, endothelial populations, and adaptive immune compartments as major contributors to plaque biology [18]. These findings have substantially expanded current understanding of vascular inflammation and cellular plasticity during atherogenesis [18,19].

Despite these advances, integration between tissue-level transcriptomic states and their cellular drivers remains limited. Bulk transcriptomic approaches provide robust pathway-level information but lack cellular resolution, whereas scRNA-seq enables detailed cellular characterization but may incompletely capture broader tissue-level biological organization. Several recent studies have attempted integration of bulk and single-cell plaque transcriptomics using deconvolution approaches; however, comparatively less attention has been directed toward defining coordinated pathway-level transcriptional states that span multiple cellular compartments [17,20]. As a result, the relationship between inflammatory and structural tissue programs and their underlying cellular ecosystems remains incompletely understood.

An additional challenge in plaque transcriptomics is the limited availability of datasets simultaneously containing paired stable and unstable lesions with high-resolution single-cell profiling from the same vascular territory. Consequently, many integrative analyses require combining datasets derived from distinct vascular beds. While carotid and coronary plaques exhibit important anatomical and hemodynamic differences, prior single-cell atlas studies suggest that core inflammatory and fibrotic transcriptional programs are broadly conserved across vascular territories, supporting exploratory cross-resolution integration approaches [13,17,18,21,22].

Emerging evidence suggests that plaque behavior may be more accurately understood through biologically distinct transcriptional endotypes rather than isolated molecular pathways alone. In this context, an endotype refers to a disease subtype defined by a distinct underlying biological mechanism [23]. Because the present study compares paired stable and unstable plaque regions rather than stratifying patients, we use the term to describe candidate transcriptional endotypes associated with plaque phenotype rather than established patient-level disease subtypes. We hypothesized that plaque phenotype reflects the relative dominance of competing inflammatory and structural transcriptional programs that can be resolved across tissue-level and cellular transcriptomic layers.

**Figure 1:**
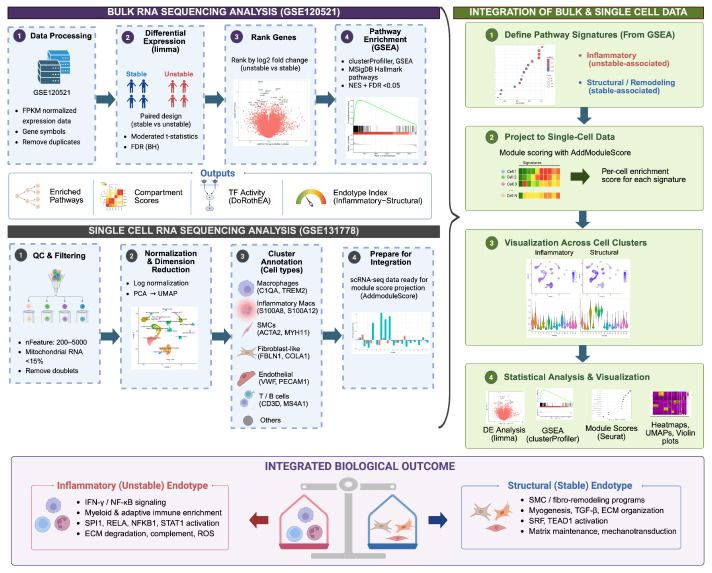
Integrated bulk and single-cell transcriptomic workflow for identifying molecular endotypes of human atherosclerotic plaque. Bulk RNA-seq data (GSE120521) were analyzed using differential expression and GSEA to identify pathways associated with stable and unstable plaques. Key pathway signatures were projected onto single-cell RNA-seq data (GSE131778) using Seurat module scoring to define their cellular origins. Integrated analysis revealed an inflammatory (unstable) endotype characterized by immune activation and macrophage-associated programs, and a structural (stable) endotype characterized by smooth muscle cell, fibro-remodeling, and extracellular matrix maintenance pathways. Abbreviations: DE, differential expression; ECM, extracellular matrix; FDR, false discovery rate; FPKM, fragments per kilobase of transcript per million mapped reads; GSEA, Gene Set Enrichment Analysis; IFN-γ, interferon gamma; NF-κB, nuclear factor kappa B; PCA, principal component analysis; QC, quality control; RNA-seq, RNA sequencing; SMC, smooth muscle cell; TF, transcription factor; UMAP, Uniform Manifold Approximation and Projection.

In the present study, we integrated paired bulk RNA sequencing data from human carotid plaques with scRNA-seq data from human coronary atherosclerotic lesions to characterize coordinated inflammatory and structural plaque transcriptional states. Using pathway enrichment analysis, cross-resolution module projection, compartment-level transcriptional scoring, and transcription factor activity inference, we sought to define the cellular and regulatory architecture underlying plaque phenotype and to establish a systems-level framework for inflammatory and structural plaque endotypes (Figure 1). Our analyses identified distinct inflammatory and structural transcriptional states associated with unstable and stable plaque phenotypes, respectively, and linked these programs to specific cellular compartments and regulatory networks. These findings provide a biologically informed framework for understanding plaque heterogeneity and support the concept of inflammatory and structural plaque endotypes in human atherosclerosis. 

## 2. Materials and Methods

### 2.1. Study Design and Public Datasets

This study employed an integrative transcriptomic framework combining bulk RNA sequencing and scRNA-seq datasets derived from human atherosclerotic plaque tissue. Publicly available datasets were obtained from the Gene Expression Omnibus (GEO). Bulk transcriptomic analysis was performed using GSE120521, which contains paired stable and unstable regions from human carotid atherosclerotic plaques. Single-cell transcriptomic analysis was performed using GSE131778, consisting of dissociated cells from human coronary atherosclerotic lesions. The study was designed to identify tissue-level transcriptional programs associated with plaque phenotype and to map these programs to specific cellular compartments and regulatory networks.

Because all datasets were obtained from publicly available de-identified repositories, additional institutional review board approval was not required. The bulk and single-cell datasets originated from different vascular beds (carotid versus coronary); therefore, the study was designed as an exploratory cross-resolution analysis intended to identify conserved transcriptional programs rather than direct one-to-one anatomical correspondence.

### 2.2. Bulk RNA Sequencing Analysis

#### 2.2.1. Data Processing and Normalization

Normalized expression matrices from GSE120521 were imported into R (version 4.5.2). Gene symbols were extracted and duplicate entries were removed by retaining the first occurrence of each gene. Expression values were log2-transformed using a pseudocount transformation: log2(FPKM + 1)

This transformation was used to stabilize variance across genes for exploratory transcriptomic analysis and preranked pathway enrichment. Because only processed FPKM-normalized matrices were publicly available through GEO, count-based normalization approaches such as voom transformation of raw counts could not be performed.

#### 2.2.2. Differential Expression Analysis

Differential expression analysis between stable and unstable plaque regions was performed using the limma package. Because stable and unstable regions were paired within the same individuals, a paired linear model was used to account for inter-patient variability: Expression ~ pair_id + condition, where condition represented stable versus unstable plaque phenotype and pair_id accounted for matched patient samples. Empirical Bayes moderation was applied using the eBayes function to improve variance estimation in the small paired cohort. Genes were ranked according to moderated t-statistics and associated *p*-values. False discovery rate (FDR) adjustment was performed using the Benjamini–Hochberg method.

#### 2.2.3. Gene Set Enrichment Analysis

Preranked gene set enrichment analysis (GSEA) was performed using clusterProfiler. Genes were ranked according to moderated t-statistics derived from limma differential expression analysis. Hallmark gene sets from the Molecular Signatures Database (MSigDB) were used as the reference pathway collection. Pathways with adjusted *p*-values < 0.1 were considered enriched. Normalized enrichment scores (NES) were used to quantify pathway activity and directionality.

### 2.3. Single-Cell RNA Sequencing Analysis

#### 2.3.1. Data Processing and Quality Control

Single-cell RNA sequencing data from GSE131778 were analyzed using Seurat. Cells with low feature counts, excessive detected features suggestive of doublets, or elevated mitochondrial transcript percentages were excluded during quality control preprocessing. Cells expressing fewer than 200 genes or greater than 5000 detected genes were removed. Cells with mitochondrial transcript content greater than 15% were also excluded. Data were log-normalized and highly variable genes were identified, and dimensionality reduction was performed using principal component analysis (PCA). Shared nearest-neighbor graph clustering was performed using the FindNeighbors and FindClusters functions with a clustering resolution of 0.6. Uniform manifold approximation and projection (UMAP) was used for visualization.

#### 2.3.2. Cell Type Annotation

Cell populations were annotated using canonical lineage markers and previously published plaque scRNA-seq atlases. Major cellular compartments identified included inflammatory macrophages, TREM2hi C1Q macrophages, contractile SMCs, fibromyocyte-like stromal cells, endothelial cells, adaptive immune populations, and fibro-remodeling stromal cells.

### 2.4. Cross-Resolution Integration and Module Scoring

Bulk-derived pathway signatures were projected onto the single-cell dataset using Seurat’s AddModuleScore function. Inflammatory signatures were derived from pathways enriched in unstable plaque, whereas structural signatures were derived from pathways enriched in stable plaque. Module scores were visualized using UMAP projections and violin plots across cellular populations.

### 2.5. Quantitative Endotype Index

To summarize the relative balance between inflammatory and structural transcriptional programs, we derived an inflammatory–structural endotype index for each plaque sample. This study-specific index was calculated as follows:

*Endotype Index = Inflammatory Score − Structural Score*

where Inflammatory and structural scores were defined as the mean expression of genes belonging to the corresponding bulk-derived pathway signatures after z-score normalization. Positive values indicate relative predominance of inflammatory transcriptional programs, whereas negative values indicate relative predominance of structural remodeling programs. This index was developed for the present study to provide a simple quantitative summary of the relative balance between the two transcriptional programs. Paired comparisons were performed using paired *t*-tests and Wilcoxon signed-rank tests.

### 2.6. Bulk Cellular Compartment Scoring

Compartment-level transcriptional scores were calculated using curated marker gene signatures representing inflammatory myeloid cells, SMC/fibro-remodeling populations, endothelial cells, and adaptive immune populations. Mean expression values for each compartment signature were compared between stable and unstable plaque regions.

### 2.7. Transcription Factor Activity Inference

Regulatory network activity was inferred using decoupleR with the DoRothEA transcription factor regulon database. High-confidence human regulons (confidence levels A–C) were used for transcription factor activity inference. Univariate linear modeling (ULM) was applied to estimate transcription factor activity scores from bulk transcriptomic profiles.

### 2.8. Statistical Analysis and Data Visualization

All analyses were performed in R (version 4.5.2). Major packages included limma, clusterProfiler, Seurat, decoupleR, DoRothEA, tidyverse, ggplot2, pheatmap, enrichplot, and ComplexHeatmap. Statistical significance was defined using two-sided testing.

All datasets analyzed in this study are publicly available through GEO under accession numbers GSE120521 and GSE131778. Computational analyses were performed using reproducible open-source workflows implemented in R. Analysis scripts will be made available upon reasonable request.

## 3. Results

### 3.1. Bulk Transcriptomic Analysis Reveals Coordinated Inflammatory Programs in Unstable Plaque

**Figure 2:**
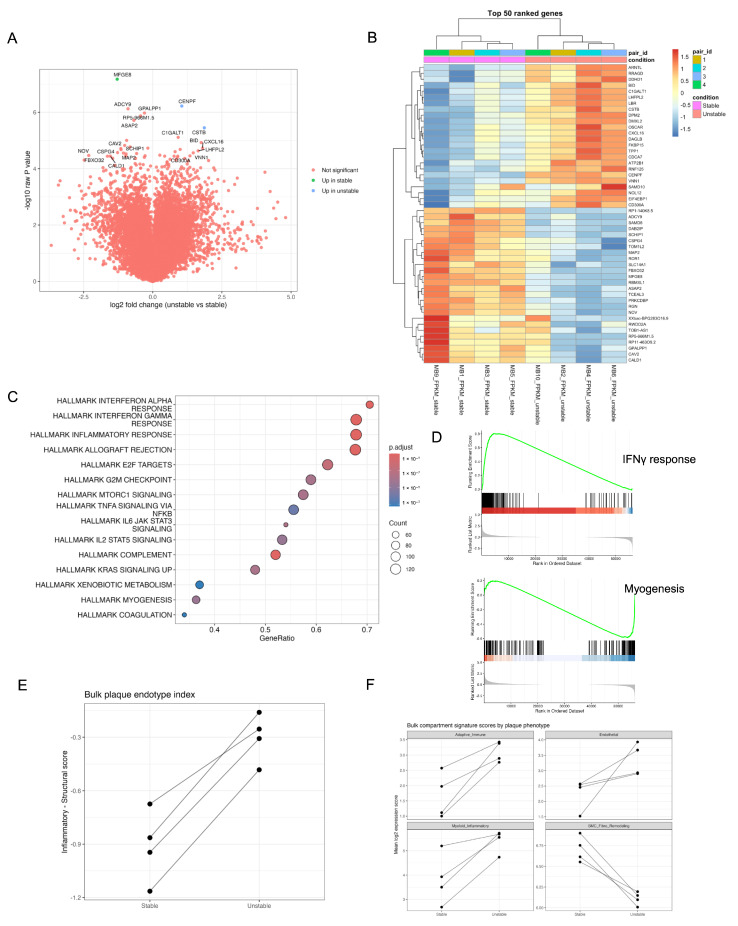
Bulk transcriptomic characterization of stable and unstable atherosclerotic plaques. (**A**) Principal component analysis of paired stable and unstable plaque samples demonstrating partial separation by plaque phenotype despite substantial inter-patient variability. (**B**) Differential gene expression analysis comparing stable and unstable plaques. Representative genes associated with unstable plaque included *CXCL16*, *CSTB*, and *CENPF*, whereas *MFGE8* and *CALD1* were enriched in stable plaque. (**C**) Hallmark gene set enrichment analysis showing inflammatory pathways enriched in unstable plaques and structural/remodeling pathways enriched in stable plaques. (**D**) Representative GSEA enrichment plots illustrating inflammatory transcriptional programs in unstable plaque and structural remodeling programs in stable plaque. (**E**) Paired inflammatory–structural endotype index across matched plaque samples. Unstable plaques demonstrated a consistent shift toward inflammatory dominance relative to paired stable regions. (**F**) Compartment-level transcriptional scores showing enrichment of myeloid and adaptive immune programs in unstable plaques and smooth muscle cell/fibro-remodeling programs in stable plaques.

To define transcriptional differences between stable and unstable atherosclerotic plaque regions, we analyzed paired bulk RNA sequencing data from human carotid plaques (GSE120521). PCA demonstrated partial separation between stable and unstable plaque regions, although inter-patient variability remained a major source of overall transcriptional variance (Figure 2A).

Differential expression analysis identified relatively few genes reaching significance after multiple testing correction, consistent with the limited statistical power of the small paired cohort. Nevertheless, several biologically relevant genes demonstrated differential expression between plaque phenotypes, including CXCL16, CSTB, and CENPF in unstable plaque and MFGE8 and CALD1 in stable plaque (Figure 2B, Table S1). These findings suggested that plaque phenotype may be driven less by isolated genes and more by coordinated transcriptional programs.

To evaluate pathway-level differences, we performed preranked Hallmark gene set enrichment analysis. Unstable plaque demonstrated enrichment of inflammatory and immune-associated pathways, including interferon gamma response, inflammatory response, interferon alpha response, TNFα signaling via NF-κB, IL6/JAK/STAT3 signaling, complement activation, and reactive oxygen species(ROS) signaling (Figure 2C, Table S2). In contrast, stable plaque regions demonstrated relative enrichment of myogenesis, epithelial–mesenchymal transition, and TGF-β–associated structural remodeling programs. Representative GSEA enrichment plots demonstrated clear separation of inflammatory (IFN-γ response) versus structural (myogenesis) transcriptional states across plaque phenotypes (Figure 2D).

To quantify the relative balance between inflammatory and structural transcriptional programs, we calculated an inflammatory–structural endotype index for each plaque sample. Across all four matched patient pairs, unstable plaque regions demonstrated a consistent shift toward inflammatory dominance relative to paired stable regions (Figure 2E).

Compartment-level transcriptional scores were calculated using curated marker signatures. Unstable plaques demonstrated enrichment of myeloid inflammatory and adaptive immune programs, whereas stable plaques demonstrated higher SMC/fibro-remodeling scores (Figure 2F).

Collectively, bulk transcriptomic analyses demonstrated that unstable plaques are characterized by coordinated inflammatory pathway activation and immune-cell enrichment, whereas stable plaques retain structural remodeling programs associated with smooth muscle cell biology. 

### 3.2. Single-Cell RNA Sequencing Identifies Distinct Cellular Compartments Within Human Atherosclerotic Plaque

**Figure 3:**
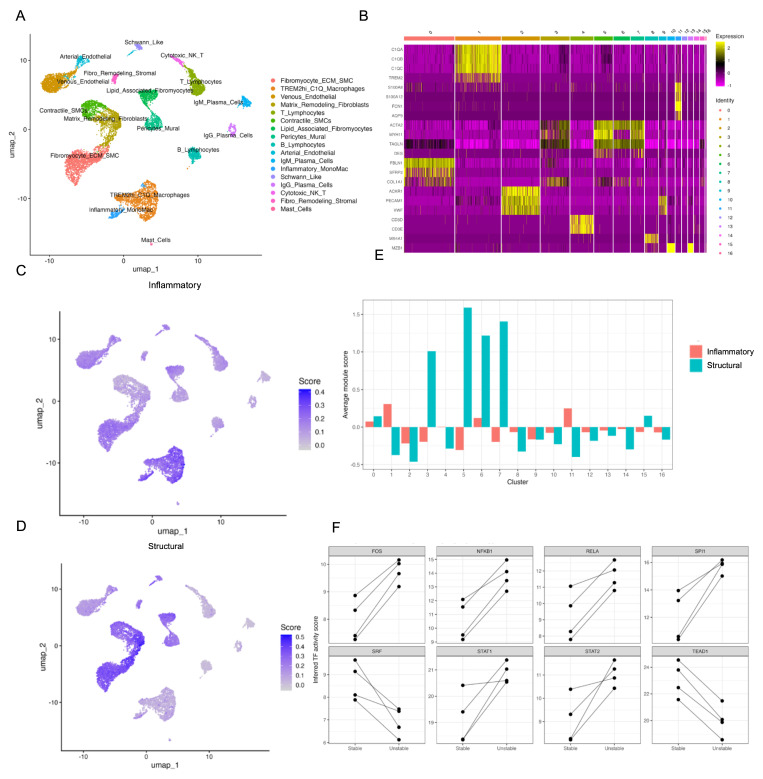
Single-cell transcriptomic analysis and cellular localization of plaque-associated transcriptional programs. (**A**) UMAP visualization of human atherosclerotic plaque scRNA-seq data demonstrating distinct cellular populations. (**B**) Canonical marker expression used for cell-type annotation. (**C**) Projection of bulk-derived inflammatory signatures onto single-cell data showing enrichment within macrophage populations. (**D**) Projection of bulk-derived structural signatures demonstrating localization within smooth muscle cell and fibromyocyte-like populations. (**E**) Quantitative comparison of inflammatory and structural module scores across cell populations. (**F**) Differential transcription factor activity analysis identifying inflammatory regulators (SPI1, NFKB1, RELA, STAT1) associated with unstable plaque and structural regulators (SRF, TEAD1) associated with stable plaque.

To define the cellular basis of these transcriptional programs, we analyzed single-cell RNA sequencing data from human coronary atherosclerotic lesions (GSE131778). Unsupervised clustering identified multiple transcriptionally distinct cellular populations within plaque tissue (Figure 3A).

Canonical marker gene expression was used to annotate major plaque cell populations, including TREM2hi macrophages, inflammatory macrophages, smooth muscle cells, fibromyocyte-like stromal cells, endothelial cells, and adaptive immune populations (Figure 3B, Table S3).

Major myeloid populations included TREM2hi C1Q macrophages expressing C1QA, C1QB, C1QC, and TREM2, as well as inflammatory monocyte-derived macrophages characterized by S100A8, S100A12, FCN1, and AQP9 expression. Structural and stromal compartments included contractile smooth muscle cells expressing MYH11, ACTA2, TAGLN, and DES, fibromyocyte-like extracellular matrix remodeling cells expressing FBLN1 and SFRP2, and fibro-remodeling stromal cells enriched for THBS4 and COL9A3. Additional populations included endothelial cells, pericyte-like mural cells, T lymphocytes, B cells, plasma cells, natural killer cells, Schwann-like cells, and mast cells.

These findings demonstrated substantial cellular heterogeneity within human atherosclerotic plaque and supported the existence of distinct inflammatory and structural cellular compartments.

### 3.3. Projection of Bulk-Derived Pathway Signatures Reveals Distinct Cellular Endotype Programs

To connect tissue-level transcriptional programs with cellular populations, inflammatory and structural signatures derived from bulk RNA sequencing analysis were projected onto the single-cell dataset using module scoring. Inflammatory signatures derived from unstable plaque demonstrated enrichment within TREM2hi macrophage and inflammatory monocyte/macrophage clusters, whereas structural signatures localized predominantly to smooth muscle cell and fibromyocyte-like stromal populations (Figure 3C,D).

Quantitative comparison of module scores across cellular clusters demonstrated that inflammatory module activity was highest within inflammatory myeloid populations, whereas structural module scores were enriched in SMC and fibro-remodeling compartments (Figure 3E, Table S4).

These findings support a model in which plaque phenotype reflects the relative dominance of inflammatory myeloid versus structural stromal transcriptional programs. Because these analyses integrated carotid bulk transcriptomics with coronary single-cell datasets, the observed localization patterns should be interpreted as evidence of conserved atherosclerotic transcriptional states rather than direct anatomical correspondence across vascular beds.

### 3.4. Transcription Factor Activity Analysis Identifies Distinct Regulatory Programs Associated with Plaque Phenotype

To infer upstream regulatory mechanisms underlying plaque-associated transcriptional programs, transcription factor activity analysis was performed using decoupleR and DoRothEA regulon inference. Unstable plaque regions demonstrated increased inferred activity of multiple inflammatory and myeloid-associated transcription factors, including SPI1 (PU.1), NFKB1, RELA, STAT1, STAT2, FOS, ETS1, and BATF (Figure 3F, Table S5).

Notably, SPI1 demonstrated the strongest differential activation in unstable plaque, consistent with enhanced myeloid lineage and macrophage-associated regulatory activity. Increased RELA and NFKB1 activity supported activation of NF-κB inflammatory signaling, while elevated STAT1 and STAT2 activity aligned with interferon-associated transcriptional programs identified via GSEA. In contrast, stable plaque regions demonstrated relatively higher inferred activity of TEAD1 and SRF.

### 3.5. Integrated Multi-Resolution Analysis Supports Distinct Plaque Endotypes

**Figure 4:**
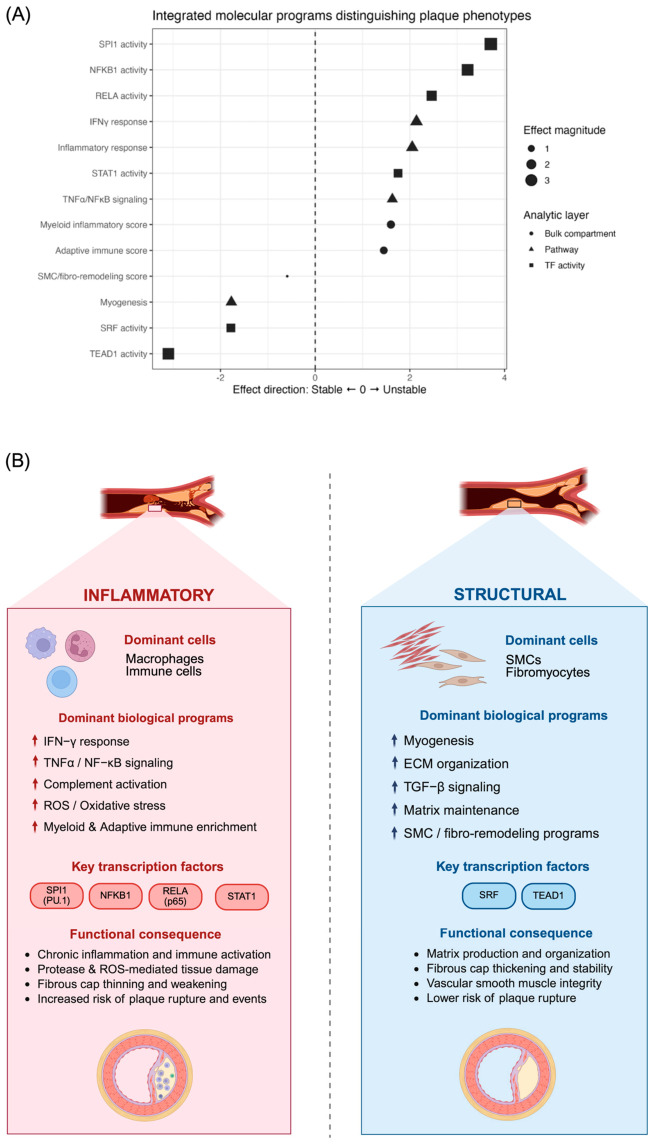
Integrated plaque endotypes. (**A**) Integrated summary of pathway enrichment, compartment-level scoring, and transcription factor activity distinguishing stable and unstable plaques. Unstable plaques were characterized by enrichment of inflammatory pathways, immune-cell programs, and increased SPI1, NFKB1, RELA, and STAT1 activity, whereas stable plaques demonstrated enrichment of myogenesis, SMC/fibro-remodeling programs, and increased SRF and TEAD1 activity. (**B**) Conceptual model of plaque endotypes. The inflammatory endotype is associated with macrophage-driven immune activation, interferon/NF-κB signaling, and plaque instability, whereas the structural endotype is characterized by smooth muscle cell and fibromyocyte-associated remodeling programs, extracellular matrix maintenance, and plaque stability.

To integrate findings across transcriptomic, cellular, and regulatory levels, we combined pathway enrichment, compartment-level scoring, module projection, and transcription factor activity analyses into a unified framework (Figure 4A).

Across multiple analytic layers, unstable plaque was consistently associated with inflammatory signaling pathways, immune-cell enrichment, and activation of macrophage-associated transcription factors, whereas stable plaque demonstrated enrichment of structural remodeling programs and smooth muscle cell–associated regulatory activity.

Based on these convergent findings, we developed a conceptual model of inflammatory and structural plaque transcriptional states that may represent candidate plaque endotypes (Figure 4B). The inflammatory endotype is characterized by macrophage predominance, activation of interferon and NF-κB signaling pathways, immune-cell enrichment, and inflammatory transcription factor activity, whereas the structural endotype is characterized by smooth muscle cell and fibromyocyte-associated remodeling programs, extracellular matrix maintenance, and structural regulatory networks.

Collectively, these results support a systems-level framework in which unstable plaque is associated with inflammatory myeloid activation, whereas stable plaque is associated with preservation of structural remodeling programs.

## 4. Discussion

In this study, we integrated paired bulk RNA sequencing and single-cell transcriptomic analyses to characterize inflammatory and structural transcriptional programs associated with human atherosclerotic plaque phenotype. Our findings support a systems-level framework in which unstable plaque is characterized by inflammatory myeloid activation and interferon/NF-κB signaling, whereas stable plaque demonstrates enrichment of SMC-associated remodeling programs.

Many of the individual inflammatory and structural pathways identified in this study have been described previously. Rather than establishing new biological associations, the primary contribution of our work is the integration of tissue level, cellular, and regulatory transcriptomic analyses into a unified framework. One of the principal findings was the enrichment of inflammatory pathways in unstable plaque, including IFN-γ response, TNFα/NF-κB signaling, IL6/JAK/STAT3 signaling, complement activation, and ROS pathways. These observations are consistent with prior studies demonstrating that inflammatory cytokine signaling promotes macrophage activation, extracellular matrix degradation, oxidative stress, and fibrous cap weakening during plaque progression [8,24]. Interferon-associated signaling has been linked to enhanced macrophage inflammatory activation and matrix metalloproteinase production, while NF-κB signaling functions as a major regulator of vascular inflammation and innate immune activation [9,24,25,26,27,28]. The convergence of these pathways across both bulk and single-cell analyses suggests that plaque instability reflects a coordinated inflammatory transcriptional state rather than activation of isolated pathways alone. This concept is further supported by the Canakinumab Anti-inflammatory Thrombosis Outcomes Study (CANTOS), which demonstrated that inhibition of IL-1β with canakinumab reduced recurrent cardiovascular events independent of lipid lowering, providing clinical evidence that targeting inflammation can modify atherosclerotic disease progression [27]. Single-cell analyses localized inflammatory programs predominantly to TREM2hi macrophage and inflammatory monocyte-derived macrophages. Recent studies have identified TREM2-expressing macrophages as a major component of advanced human atherosclerotic lesions [14,15]. Although the biological role of TREM2 appears context dependent, these cells likely represent metabolically active populations involved in lipid handling, inflammatory signaling, and tissue remodeling [29,30,31,32,33]. In parallel, inflammatory monocyte-derived macrophages expressing S100A8, S100A12, and FCN1 likely represent a more acutely inflammatory compartment associated with innate immune activation and cytokine signaling [14,34]. Together, these findings support the growing recognition that macrophage heterogeneity plays a central role in determining plaque phenotype.

In contrast, stable plaque demonstrated relative enrichment of myogenesis, extracellular matrix(ECM) organization, and TGF-β associated remodeling pathways together with increased SRF and TEAD1 activity. The SRF–myocardin axis is essential for maintenance of contractile SMC phenotype and vascular wall integrity [10,35]. Loss of SMC contractile identity and transition toward modulated inflammatory or fibromyocyte-like states are increasingly recognized as central features of progressive atherosclerosis [11,36]. While TEAD1 has been implicated in vascular development and mechanotransduction, its role in atherosclerosis appears more complex and may reflect broader Hippo pathway activity and SMC phenotypic plasticity rather than a purely contractile state [37,38]. These findings underscore the dynamic nature of structural remodeling programs within atherosclerotic lesions.

An important aspect of this study was the integration of tissue-level transcriptomic programs with cellular localization. While previous studies have used deconvolution or clustering approaches to characterize plaque heterogeneity, our analysis focused on coordinated pathway-level transcriptional states spanning multiple cellular compartments [21]. Spatial transcriptomic analyses have further demonstrated regional enrichment of immune and extracellular matrix programs within rupture-prone plaque regions [39,40]. This framework supports the concept of inflammatory and structural plaque transcriptional states that may represent candidate endotypes defined by distinct underlying biological mechanisms rather than morphology alone.

The inflammatory–structural endotype index provided additional support for this framework. Although statistical interpretation was necessarily limited by the very small paired bulk cohort (*n* = 4), these analyses should be considered exploratory and hypothesis-generating and all matched plaque pairs demonstrated the same directional shift toward inflammatory enrichment in unstable lesions. While directional consistency alone cannot substitute for statistical power, the concordance across all pairs supports the biological coherence of the observed transcriptional pattern and aligns with the broader pathway-level findings from the study.

Transcription factor activity analyses further supported the existence of distinct regulatory programs underlying plaque phenotype. Increased inferred activity of SPI1 (PU.1), RELA, NFKB1, STAT1, and STAT2 in unstable plaque aligned closely with the inflammatory pathways identified via GSEA and with macrophage-associated cellular localization observed in the single-cell analyses. PU.1 is a master regulator of myeloid lineage differentiation and macrophage transcriptional identity, functioning as a lineage-determining transcription factor within macrophages [41,42]. Recent multi-omics studies have demonstrated STAT1–PU.1 co-binding at promoters of interferon-responsive macrophage genes in atherosclerotic lesions, providing a mechanistic link between interferon signaling and macrophage inflammatory activation [43]. Conversely, higher inferred activity of SRF and TEAD1 in stable plaque was consistent with enrichment of vascular remodeling and mechanotransduction-associated programs [36,37]. Together, these findings suggest that plaque phenotype is associated not only with distinct cellular populations but also with coordinated upstream regulatory networks.

Several limitations should be acknowledged. First, the bulk RNA sequencing cohort was limited to 4 paired stable and unstable plaque samples, substantially restricting statistical power and limiting the robustness of gene-level differential expression analyses. To mitigate this limitation, we focused primarily on coordinated pathway-level enrichment patterns and cross-resolution biological consistency rather than isolated gene-level findings. Second, the bulk and single-cell datasets were derived from different vascular beds, with carotid tissue used for bulk transcriptomics and coronary lesions used for scRNA-seq analysis. Although inflammatory and fibrotic pathways are broadly conserved across vascular territories, recent studies have demonstrated important vascular bed-specific differences in macrophage metabolic programs and transcriptional organization, suggesting that plaque biology may not be fully interchangeable across anatomical sites [13,22,44]. Third, transcriptomic analyses reflect gene-expression states rather than direct protein-level activity or functional behavior, and the inferred transcription factor activity results were computationally derived rather than experimentally validated. Finally, the study represents a single temporal snapshot of plaque biology and therefore cannot capture the dynamic processes of plaque progression, destabilization, and healing overtime. Fifth, publicly available datasets were generated using different tissue-processing methods, sequencing platforms, and quality-control pipelines, potentially introducing systematic integration bias. Finally, module scoring approaches such as AddModuleScore may introduce biases related to transcript detection variability and signature composition, and pathway projection from bulk to single-cell resolution should therefore be interpreted cautiously.

Despite these limitations, the study has several strengths. The paired bulk design reduced inter-individual variability and enabled direct comparison of stable and unstable plaque regions within the same patients. Integration of pathway enrichment analysis, compartment scoring, single-cell localization, and transcription factor inference provided convergent evidence across multiple analytic layers. In addition, the focus on coordinated biological programs rather than isolated genes aligns with the increasingly recognized systems-level complexity of atherosclerosis.

Future studies should seek validation in larger cohorts incorporating matched bulk, single-cell, and spatial transcriptomic profiling from the same vascular territories. Recent spatial transcriptomic studies have already begun to reveal highly organized plaque microenvironments, including spatial gradients of macrophage differentiation, fibroblast-like SMC signaling niches associated with plaque stability, and sex-specific mechanisms of plaque destabilization [39,40,45]. Integration of transcriptomic endotypes with circulating biomarkers, imaging phenotypes, and systemic inflammatory drivers such as clonal hematopoiesis may further improve understanding of plaque heterogeneity and cardiovascular risk stratification [21]. Additional investigation will also be needed to determine whether these inflammatory and structural transcriptional states are associated with differential therapeutic responses.

## 5. Conclusions

In conclusion, our integrative transcriptomic analysis supports a conceptual framework of inflammatory and structural plaque transcriptional states that may represent candidate endotypes in human atherosclerosis. Unstable plaque was characterized by inflammatory myeloid dominance, interferon/NF-κB regulatory activation, and immune-associated transcriptional enrichment, whereas stable plaque demonstrated relative preservation of vascular remodeling and extracellular matrix-associated programs. These findings provide a systems-level framework linking tissue-level plaque phenotype with underlying cellular and regulatory architecture and support the concept of inflammatory and structural plaque endotypes in human atherosclerosis.

## Data Availability

The datasets analyzed in this study are publicly available in the GEO under accession numbers GSE120521 and GSE131778.

## Supplementary Materials

The following supporting information can be downloaded at https://www.mdpi.com/article/10.3390/genes17070779/s1. Table S1: Differentially expressed genes between stable and unstable plaque regions; Table S2: Hallmark gene set enrichment analysis results; Table S3: Canonical marker genes used for single-cell cluster annotation; Table S4: Cell population-specific inflammatory and structural module scores; Table S5: Differential transcription factor activity inference results.

## Abbreviations

The following abbreviations are used in this manuscript:

ACS	Acute Coronary Syndrome
ECM	Extracellular Matrix
FDR	False Discovery Rate
FPKM	Fragments Per Kilobase of Transcript per Million Mapped Reads
GEO	Gene Expression Omnibus
GSEA	Gene Set Enrichment Analysis
IFN-γ	Interferon Gamma
IL6	Interleukin 6
MSigDB	Molecular Signatures Database
NES	Normalized Enrichment Score
NF-κB	Nuclear Factor Kappa B
PCA	Principal Component Analysis
QC	Quality Control
RNA-seq	RNA Sequencing
ROS	Reactive Oxygen Species
scRNA-seq	Single-Cell RNA Sequencing
SMC	Smooth Muscle Cell
STAT	Signal Transducer and Activator of Transcription
TF	Transcription Factor
TGF-β	Transforming Growth Factor Beta
TNFα	Tumor Necrosis Factor Alpha
ULM	Univariate Linear Model
UMAP	Uniform Manifold Approximation and Projection
